# Enhanced characterisation for the management of industrial steel processing by products: potential of sequential chemical extraction

**DOI:** 10.1007/s10661-019-7275-9

**Published:** 2019-02-27

**Authors:** Kiri J. Rodgers, Iain S. McLellan, Simon J. Cuthbert, Andrew S. Hursthouse

**Affiliations:** 1 School of Science & Sport, University of the West of Scotland, Paisley, PA1 2BE UK; 2 Hunan Regional Key Laboratory for Shale Gas Resource Exploitation, Xiangtan, Hunan Province China

**Keywords:** Sequential chemical extraction, Metal process by products, Waste management, Hazardous waste

## Abstract

There is a pressing need for innovative waste management approaches as environmental regulations become more stringent worldwide alongside increasing demand for a more circular economy. Sequential chemical extraction (SE) analysis, which has previously been applied to environmental media such as soils and sediments, offers the potential to provide an understanding of the composition of solid steel processing by products, aiding the waste classification process and improving environmental protection. The definition of seven-phase associations through a SE method evaluated in this study were for (1) water soluble, (2) ion exchangeable, (3) carbonate, (4) amorphous Fe–Mn oxides, (5) crystalline Fe–Mn oxides, (6) sulphides and (7) silicate residues. Steel waste by-products (flue dust and filter cake) were evaluated for both extracted components (ICP analysis) and residual phases (using powder X-ray diffraction, SEM and FTIR), to model the transformations taking place during extraction. The presence and removal of important potentially toxic element (PTE) host solid phases were confirmed during extraction. The SE protocol provides key information, particularly for the association of potentially toxic elements with the first three extracts, which are most sensitive in waste management processes. The water-soluble phase is the most available followed by ion-exchangeable and carbonate fractions, all representing phases more sensitive to environmental change, in particular to pH. This study demonstrates that the distribution of potentially toxic elements such as zinc, lead and copper between sensitive and immobile phases can be reliably obtained in technological process by-products. We demonstrate that despite heterogeneity as a major variable, even for fine particulate matter, SE can provide more refined classification with information to identify reuse potential and ultimately minimise hazardous waste streams.

## Introduction

Waste management regulations worldwide are becoming more stringent, and the option to landfill wastes is being increasingly reduced (CEWEP 2012; Cointreau 2008; World Steel Association 2014). As a result, industrial operators from steel production or mining are faced with a critical need to assess their waste generation and the sustainability of current production processes. The amount of waste being generated can corrupt the production process infrastructure and can also cause environmental damage and breach regulatory thresholds. This adds to financial operating costs and causes us to question the sustainability of the production processes. The core aim of this study was to investigate the potential of chemical extraction procedures to improve waste characterisation and evaluate implications to improve management practice within the steel industry life cycle. Sequential chemical extraction (SE), which is a technique previously widely applied to study metal associations within soils and sediments, is utilised to fractionate the chemical composition of solid wastes in order to provide material characterisation in a robust approach and an alternative to the application of multiple analytical techniques, e.g. SEM, XRD, pseudo-total digestions and FTIR. In addition, the opportunity to learn more about elemental associations supports the development of sustainable processes and a more circular economy within the steel industry: through stabilisation and reclassification of commercially valuable elements which could be re-used within the production line, reducing waste and therefore costs to an industrial operation.

### Sequential extraction

Sequential chemical extractions are widely used to operationally classify metals and potentially toxic elements (PTEs) in solid phases by their associations with discrete extraction phases or reactive fractions in solids media. Additionally, these extractions can be used as models to simulate various environmental scenarios, offering an insight to reactivity if material exposure occurs to sensitive receptors or surrounding conditions change with implications for wider environmental risk.

The two most commonly applied SE procedures are referred to as the BCR (Community Bureau of Reference) (Bacon et al. 2005) and Tessier (Tessier et al. 1979); however, both procedures have limitations when applied to industrial wastes, e.g. the BCR method has been reported to show misclassification for Zn during the first step and Cu in the second step when compared to certified materials for slags (Prudent et al. 1996), sediments (Zhan and Guo 2013, Cantario et al. 2012) and mining waste (Bacon and Davidson 2008), whereas Tessier’s procedure requires constant modifications dependent on sample matrix particularly with regard to pH (Filgueiras et al. 2002). Although there is some bias towards these operating procedures, the application of SE has not been widely assessed for industrial wastes even though its potential has been demonstrated (Rodgers et al. 2015). The value of SE lies in its efficiency to characterise the mobility of PTEs in waste that in turn determines hazard potential which can then be assessed for future treatment/stabilisation options for materials. Previous studies have identified key characteristics of technological materials such as high proportion of metal constituents (Bacon and Davidson 2008) particle size and pH variation which can provide opportunities for comparative use of SE. For example in relation to mine wastes, Leinz et al. (2000) developed a seven-step series of extractions associated with operationally defined phases: water soluble, ion exchangeable, carbonate, amorphous Fe-oxide, crystalline Fe-oxide, sulphide and silicate.

This approach is particularly relevant for wastes from steel manufacturing, where the ability to distinguish between amorphous and crystalline Fe–Mn oxides and sulfide bounds forms which exhibit varying reactivity and environmental sensitivity given our current understanding of phase associations of PTEs in these waste materials presents useful fractionation information supporting waste management options. Furthermore, this can help reduce overly conservative allocation of waste to hazardous categories and improve sustainability of industrial operations.

In this study, we evaluated the seven-step methodology applied to a number of typical steel production by-products. These include flue dust (FD) produced in significant quantities from dust-laden fumes of the steel-making process which utilise large amounts of oxygen in production. For example, dust can be expelled from the top of the blast furnace during smelting or during compaction in the sintering process. These dusts are collected using a range of techniques and leads to the generation by-product from the basic oxygen furnace (BOF) and the filter cake (FC), which is the material produced from the filtering process in gas cleaning systems.

As previous experimental studies have shown procedural adjustments may be required to adapt to the different physico-chemical properties of steel wastes (Rodgers et al. 2015), the potential of SE was explored and a method validation exercise undertaken to provide practical assessment of application. The approach described here uses additional solid-phase analysis (SPA) techniques, e.g. solid-phase identification where suitable by X-ray diffraction (XRD), elemental distribution in prepared samples by scanning electron microscopy (SEM) and active functional groups—particularly metal oxides, carbonates and other carbon-containing functional groups by Fourier transform infrared spectroscopy (FTIR), to provide various characteristic information, e.g. *key* functional groups or mineral phases, to model the PTE partitioning and extraction during the application of the seven-step protocol.

## Experimental

### Sequential extraction

Samples were supplied from a steel company, air dried, sieved to less than 2 mm, mixed, cone and quartered (IUPAC 2017) before being weighed out for experimentation (Table 1). The theoretical reactivity is briefly summarised in a table of predicted chemical reactivity during each step (Table 2), providing context for assessment of each extraction step and interpretation of data from solid and liquid phases.

**Table 1:** Summary of sequential extraction method

Step	Phase	Reagent	Description of procedure
1	Water soluble	0.25 g sample, with 0.25 g silica gel and 25 ml deionised water	Reagents mixed in a 50 ml centrifuge tube for 2 h in a horizontal, reciprocating shaker.
2	Ion exchangeable	25 ml 1 M sodium acetate	Residue extracted for 1 h in a horizontal reciprocating shaker, at ambient temperature.
3	Carbonate associated	25 ml 1 M sodium acetate buffered to pH < 5 with acetic acid	Shaken for 2 h
4	FeOX_am_	25 ml hydroxylamine hydrochloride in 0.25 M HCl	Residue is extracted for 30 min in a water bath at 50 °C
5	FeOX_cryst_	25 ml 4 M HCl	30 min water bath at 94 °C
6	Sulphide	1st extract: 2 g of sodium chlorate +10 ml conc HCl. Then deionised water (DI)	Initial reagents added over 45 min, extracted and then diluted to 25 ml. Residue is then extracted for a further 40mins in a boiling water bath. The two extracts are analysed separately and results are combined.
		2nd extract: 25 ml 4 M HNO_3_	
7	Residual/silicate	Aqua regia (HCl:HNO_3_)	Residue transferred to a Teflon beaker and is digested with aqua regia

**Table 2:** Predicted reactions from each extraction step

Extraction phase	Example reactivity	Possible solid contaminants in residue	Comments	Reference
Water-soluble fraction	2 KCl _[S]_ + 2 H_2_O _[l]_ → 2 KOH _[aq]_ + Cl_2 [g]_ + H_2 [g]_	Silica	This refers to the water-soluble component where salts of group 1 elements (from the periodic table) are expected to be extracted including metal chlorides. This suggests easily extracted metals from acid rain.	(Weil and Brady 2016)
Ion-exchangeable fraction	Ca (OH)_2_ + 2 CH_3_COONa → Ca^2+^ + 2OH^−^ + Na^+^ + CH_3_COO^−^	Sodium acetate, NaCl, Na-bearing silicates	This refers to the ion-exchangeable fraction which saturates anions bound to metals, which in turn is likely to release cations that can cause the release of their respective salts due to ion exchange.	(Maul et al. 2014, Lee 1999)
Carbonate fraction	CaCO_3_ + CH_3_COONa (CH_3_COOH) → Ca(C_2_H_3_O_2_)_2 [aq]_ + NaOH + CO_2_	Sodium acetate, NaCl, Na-bearing silicates, Na_2_CO_3_ (or monohydrate)	This refers to the carbonate fraction where light acidification can dissolve these carbonates as they decompose the complex releases the metals as acetate salts. These will then fully dissociate into aqueous solution.	(David and Leventhal 1995)
Amorphous Fe–Mn oxides	4Fe(OH)_3_ + 2NH_2_OH·HCl → 4 Fe (OH)_2_ + 5H_2_O + N_2_O + 2H^+^ + 2Cl^−^	Nitrates, chlorides	This refers to amorphous Fe–Mn oxides, and the extraction process depends on the redox chemistry that is taking place. The hydroxylamine hydrochloride acts as a two-electron reductant, so Fe(III) and Mn (Nobre et al.) reduced to Fe(II) and Mn(II).	(Baba and Adekola 2010)
Crystalline Fe–Mn oxides	Fe_3_O_4 [s]_ + 8 HCl _(Curiao et al.)_ → FeCl_2 (Curiao et al.)_ + 2 FeCl_3 (Curiao et al.)_ + 4 H_2_O _(l)_	Chlorides	This refers to crystalline Fe–Mn oxides where a standard acid–base reaction allows the metal oxides to react, producing metal chlorides in solution, which are then soluble.	(Baba and Adekola 2010, David and Leventhal 1995)
Sulphide fraction	2ZnS_(S)_ + 2NaClO_3(S)_ + 4HCl_(Curiao et al.)_ → 2ZnCl_2(Curiao et al.)_ + S^0^_(g)_ + Na_2_SO_4(S)_ + 2H_2_O_(l)_ + Cl_2(g)_	NaCl Nitrates (sulfates?)	This refers to sulfide components in complexes where a simple oxidation process using stronger acids to solubilise the sulphides and the second addition of acid will solubilise remaining sulfides.	(Baba and Adekola 2010),(Uçar 2009)
Silicates and residual fraction	4 HCl + 2 HNO_3_ + Fe → FeCl_4_^−^ + NO_2_ + NO + 3H_2_O	Chlorides, nitrates	This refers to residual silicates where a violent reaction that when in contact with reducing agents/easily oxidisable substances, e.g. metals, are rapidly attacked, producing either soluble chlorocomplexes or insoluble chlorides.	(Lee 1999)

### Reagents

All chemical reagents were obtained from Fisher Scientific (Loughborough, UK) or Sigma-Aldrich (Gillingham, Dorset, UK) and were of analytical grade, unless otherwise stated. Deionised water was used for all applications unless otherwise stated, in which case ultra-high purity water (ELGA Process Water, resistivity ≥ 18.2 MΩ cm^−1^) was used. An appropriate certified reference material (EURO STANDARD 877/1 steel furnace dust) (CRM) was used to check analytical accuracy for elemental determination.

### Instrumentation (ICP-AES)

Aqueous elemental concentrations were determined using inductively coupled plasma atomic emission spectrometry (ICP-AES) (Thermo iCAP 6000 series), with instrumental configuration as outlined in Tables 3 and 4. Samples in non-acidic matrices (i.e. pH > 2) were preserved until analysis by acidification using HNO_3_ (1% *v*/*v*) and were calibrated with three matrix-matched mixed elemental calibration standards (0 mg/l, 2 mg/l and 10 mg/l). Procedural blanks were prepared with each sample batch, and were treated as per the samples, all of which were run in triplicate.

**Table 3:** Instrumental configuration conditions (ICP-AES)

Parameter	ICP-AES
Matrix	Total digests and SEP extracts
Nebuliser gas	Argon
Collision cell gas flow	n/a
Power	1.40 kW
Coolant flow	12 l/min
Plasma flow	15 l/min
Nebuliser flow	1.0 l/min
Nebuliser	Glass concentric
Auxiliary flow	1.5 l/min
Viewing height	8 mm

*n/a* not available

**Table 4:** ICP-AES analytical wavelength interferences caused by metal rich processing by product components identified

Element	Wavelength (nm)			Interference	Caused by:
	1	2	3		
Al	396.147	394.396	308.211	N	–
B	249.776	208.885	–	Y	Fe—249.772 nm
Ba	233.519	455.408	–	N	–
Be	313.107	313.049	–	Y	*
Bi	223.057	190.174	–	N	–
Ca	317.938	422.666	315.888	N	–
Cd	228.798	241.436	–	Y	As—214.410 nm
Co	228.611	238.895	230.782	Y	Fe—238.863 nm
Cr	267.716	205.562	357.863	N	–
Cu	327.4	324.749	–	Y	Fe—324.7.28 nm
Fe	238.2	239.565	259.936	N	
K	766.494	–	–	N	–
Li	670.784	610.351	–	Y	Ca—610.272 nm
Mg	285.217	279.074	–	N	–
Mn	257.606	260.564	–	N	–
Na	589.583	588.983	–	Y	*
Ni	231.606	232.006	221.65	Y	Fe—232.036 nm; Mo—221.66 nm
Pb	216.996	220.354	–	Y	Fe—216.995 nm
Ti	334.937	336.114	–	Y	Ca—336.192 nm
Tl	190.801	276.787	–	N	–
V	310.233	292.407	309.313	Y	Al—309.271 nm
Zn	206.198	213.858	202.548	N	

For quality assurance and quality control (QA/QC) purposes, the most sensitive wavelengths were selected for elemental determination based on their Wohlers value (Todorov et al. 2014); following analysis, the values for each wavelength were compared to check for potential interferences. If no known interference was identified, a wavelength was visually selected based on peak shape (i.e. most Gaussian shaped) and intensity (the peak with the greatest intensity). There were noticeable difficulties when analysing for nickel in the sequential extraction procedure which were caused by interferences from not only Si at 221.6 nm but also Cr and Fe at 232 nm (Inorganic Ventures 2016), as this could not be resolved Ni was omitted from analysis. The wavelengths used for analysis were as follows: Cr 205.562 nm, Cu 327.400 nm, Mn 257.606 nm, Pb 22.354 nm and Zn 206.198 nm.

### Powder X-ray diffraction

Samples were prepared by crushing to < 20 μm then evenly filled into an aluminium sample holder (PMMA 0.49 mm diameter). Samples were analysed by a Siemens D5000 diffractometer, and the instrument was calibrated using a silica standard and was configured with a Cu Kα radiation source. X-ray diffraction patterns were collected from a 2*θ* operating range with a 1 to 75° and 0.01° s^−1^ angle interval/dwell time.

The baseline was subtracted from resulting diffractograms and smoothed before being compared with diffraction pattern libraries for phase identification, using the EVA software package (DIFFRACplus suite, Burker Corporation) and ICCD (The international centre for diffraction data 1997–2015). These subtractions were carried out as a result of characteristic “humps” being observed, caused by amorphous structures within the waste sample matrices, and from additional fluorescence that can be attributed to the Fe-rich matrix. The addition of a nickel “filter” helped to significantly reduce the amount of fluorescence and intensity of the Kβ lines generated in the spectrum (Suryanarayana and Norton 1998) improving resolution and phase identification.

### Fourier-transform infrared spectroscopy

Thermo Scientific Nicolet 6700 FTIR spectrometer was used for analysis and carried out at room temperature. Air-dried solid samples were analysed on the single reflection ATR (attenuated total reflectance) mode for its robustness and durability. The crystal area was cleaned, the background collected and then the powdered sample was placed onto the small crystal area (with the sample height less than a few millimetres) and scanned between 300 and 4000 nm for a total of 64 scans. Both raw and ground samples were analysed for comparison.

### Scanning electron microscopy

A Hitachi S4100 field emission SEM was used to run samples at 20 kV and a 15-mm working distance using a germanium detector. Typical sample preparation involved mounting a sample onto a “stub” with double sided tape; commonly, electrically conductive carbon is used or by impregnation in an epoxy resin mould. For both approaches, the samples are then coated with an electrically conductive material, i.e. carbon or gold using a sputter coater to reduce charging potential during analysis. These approaches were less successful due to excessive charging or inability to mix into an epoxy resin as a result of the samples high hydrophobic nature.

**Fig. 1:**
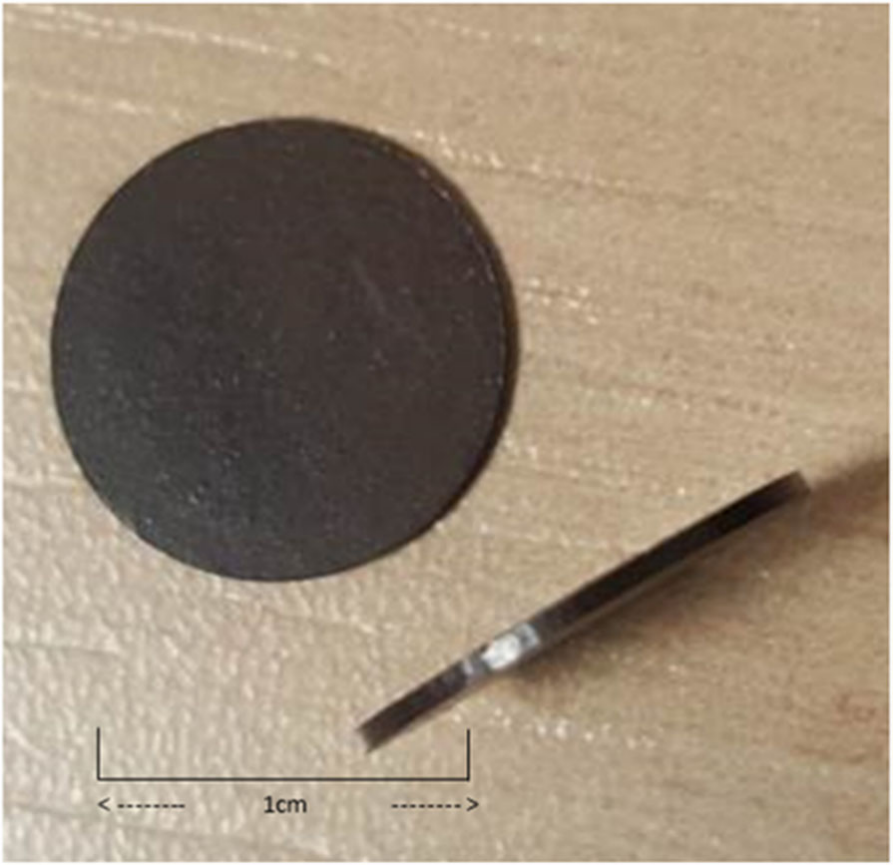
Waste samples contained in pressed KBr pellet

Therefore, the method development used to overcome these issues utilised a KBr press for the purpose of compacting the particulates together to form a pellet (Fig. 1). Each sample (approximately 200 mg–more for finer particulate) was pressed (with no addition of KBr) into a cleaned press to avoid K or Br contamination. These pellets were then additionally impregnated using an epoxy resin mixture (EPO-TEX) into moulds, where the air was removed using a Logitech impregnation Unit IU30, before being left to cure and once set the mould was cut in half and reset to obtain cross sections of the pellet.

Specimen samples were prepared in this manner to stabilise the specimen, i.e. avoid fine particle sputtering caused by charging, which is particularly problematic for the finer particles, even when being gold coated, and reduce the amount of handling of the specimen.

The majority of samples analysed were coated in gold, with a subset coated in carbon. This was because gold has a high electron output for secondary electrons and can keep the sample grounded electronically and mechanically for analysis. It needs to be acknowledged that this approach can greatly reduce the size of low energy peaks as a result of gold’s large mass and the subsequent intensity ratio. Alternatively, when carbon coating was used, a higher sensitivity for lower energy peaks was achieved; however, this can skew its own identification and charging can occur after longer analysis periods, i.e. for mapping. Both coating applications were applied to distinguish between sample compositions, particularly with regard to carbon (C) and low abundance elements.

## Results

### Pseudo-total PTE content

Initial characterisation was carried out for the two samples (FD and FC) as well as the CRM with FD and FC results shown in (Tables 5 and 6) and CRM recoveries of 96–104%. These values can be used as baseline comparisons for SE and SPA.

**Table 5:** Pseudo-total analysis results for PTEs in flue dust

	Mean (ppm)	STDEV	Mean %	Oxide	
Al	8,163.6	859.0	0.8164	3.09	*Al*_*2*_ *O*_*3*_
As	3.419	1.661	0.0003		
Ba	157.04	16.384	0.0157		
Be	0.486	0.047	0.0000		
Bi	0	0	0.0000		
Ca	694,881	72,301	69.4	97.2	*CaO*
Co	1.175	0.391	0.0001		
Cr	345.15	16.78	0.0345		
Cu	209.2	7.435	0.021		
Fe	9549	348.40	0.955	2.73	*Fe*_*2*_ *O*_*3*_
K	16,268	728.99	1.6268		
Li	89.235	3.368	0.009		
Mg	6,960.2	224.2	0.696	1.15	*MgO*
Mn	804.8	29.451	0.0805		
Na	8,000.6	305.828	0.8001	2.16	*Na*_*2*_ *O*
Ni	123.67	4.639	0.0124		
Pb	46.968	4.809	0.0047		
Sb	0	0	0.0000		
Se	436.68	30.83	0.0437		
Sr	521.8	19.91	0.0522		
Ti	31.94	2.95	0.0032		
Tl	0	0	0.0000		
V	6.562	0.444	0.0007		
Zn	45.832	1.991	0.0046		
Total			*74.66*	*106.33*	

Si, C, S, O and H were not analysed, and therefore, oxides below assume stoichiometry with oxygen and iron is presumed ferric. Elements for which the oxide exceeds 1 wt% are Al, Ca, Fe, Mg and Na. Ca is predominant at > 97% oxide, indicative of lime, Ca-silicate and/or carbonates.

**Table 6:** Pseudo-total analysis results for PTEs in filter cake

	Mean (ppm)	STDEV	Mean %	Oxide	
Al	820.51	81.478	0.0821		
As	9.57	2.283	0.0010		
Ba	43.793	4.387	0.0044		
Be	0.056	0.02	0.0000		
Bi	0	0	0.0000		
Ca	341,562	36,173	34.16	47.79	*CaO*
Co	607.227	41.485	0.0607		
Cr	38,214	3902.7	3.821	11.17	*Cr*_*2*_ *O*_*3*_
Cu	2019.6	206.67	0.202		
Fe	203,741	17,444	20.37	58.3	*Fe*_*2*_ *O*_*3*_
K	0	0	0.0000		
Li	0.352	0.032	0.0000		
Mg	2620.3	277.2	0.2620		
Mn	4658.276	490.315	0.4658		
Na	3979.2	293.35	0.397	1.07	*Na*_*2*_ *O*
Ni	36,060	3566	3.60	4.6	*NiO*
Pb	695.221	62.254	0.0695		
Sb	151.09	14.744	0.0151		
Se	4373.277	365.316	0.4373		
Sr	141.828	10.304	0.0142		
Ti	49.437	4.03	0.0049		
Tl	0	0	0.0000		
V	142.159	12.245	0.0142		
Zn	220.41	20.153	0.0220		
Total			*64.01*	*123.6*	

Si, C, S, O and H were not analysed and therefore Oxides below assume stoichiometry with oxygen and Iron is presumed ferric. Elements for which the oxide exceeds 1 wt% are Ca, Cr, Fe, Na and Ni. The oxide total is very high, so Fe is most likely in not only a ferrous form but also as the predominant cation along with Ca.

#### Sequential extraction

**Fig. 2:**
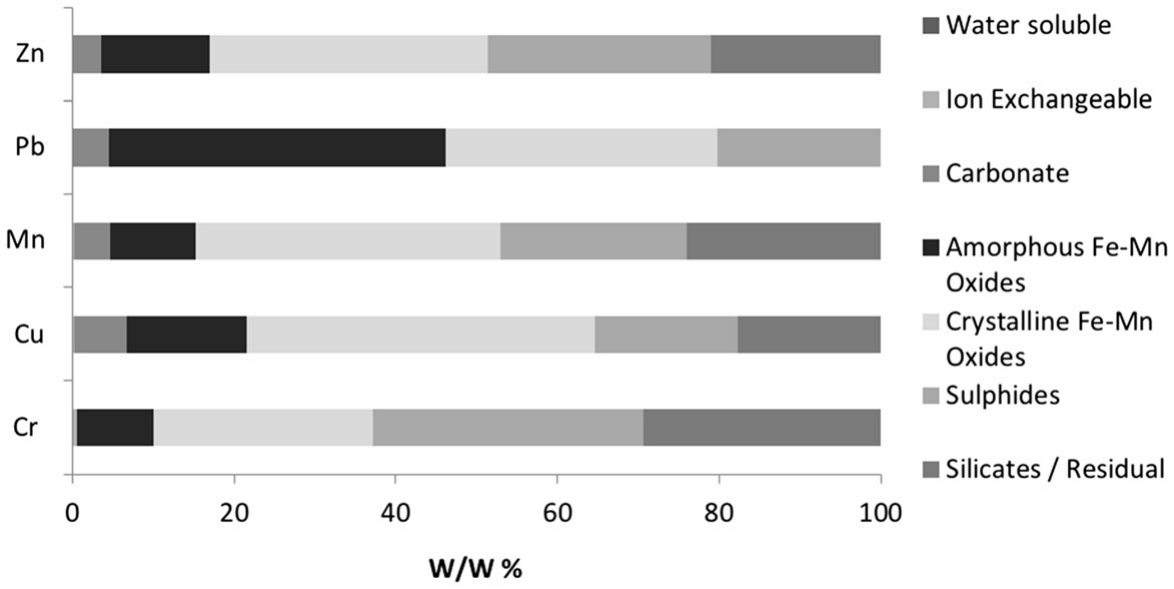
Elemental distribution in CRM 877-1 furnace dust. PTEs (normalised to 100%) across seven SE steps. Pseudo-totals: Zn 10,108 mg/kg, Pb 10,084 mg/kg, Mn 11,208 mg/kg, Cu 206.3 mg/kg, Cr 113.2 mg/kg

**Fig. 3:**
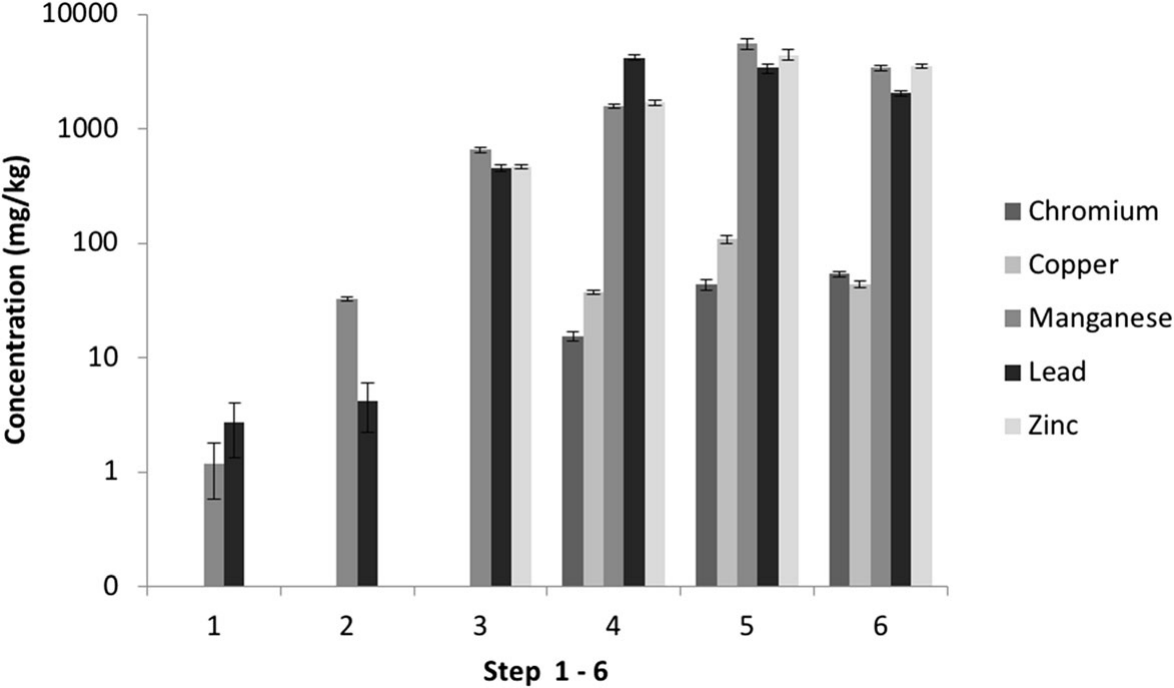
Variations between PTE concentrations from the SE extracts for CRM 877-1 furnace dust (1—water soluble, 2—ion exchangeable, 3—carbonates, 4—Fe–Mn oxides (amorphous), 5—Fe–Mn oxides (crystalline), 6—sulfides). *n* = 20. Error bars = standard deviation

**Fig. 4:**
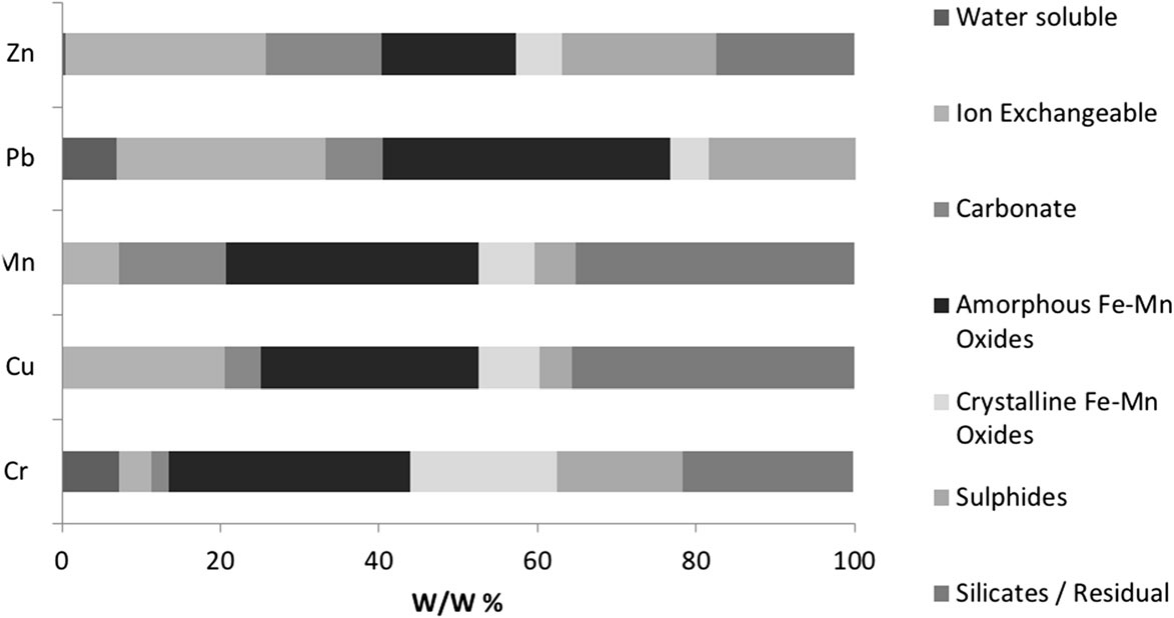
Measured elemental distribution of PTEs found in flue dust samples determined by a seven-step SEP. Pseudo-totals: Zn 67.0 mg/kg, Pb 45.8 mg/kg, Mn 803.1 mg/kg, Cu 209.2 mg/kg, Cr 343.8 mg/kg

**Fig. 5:**
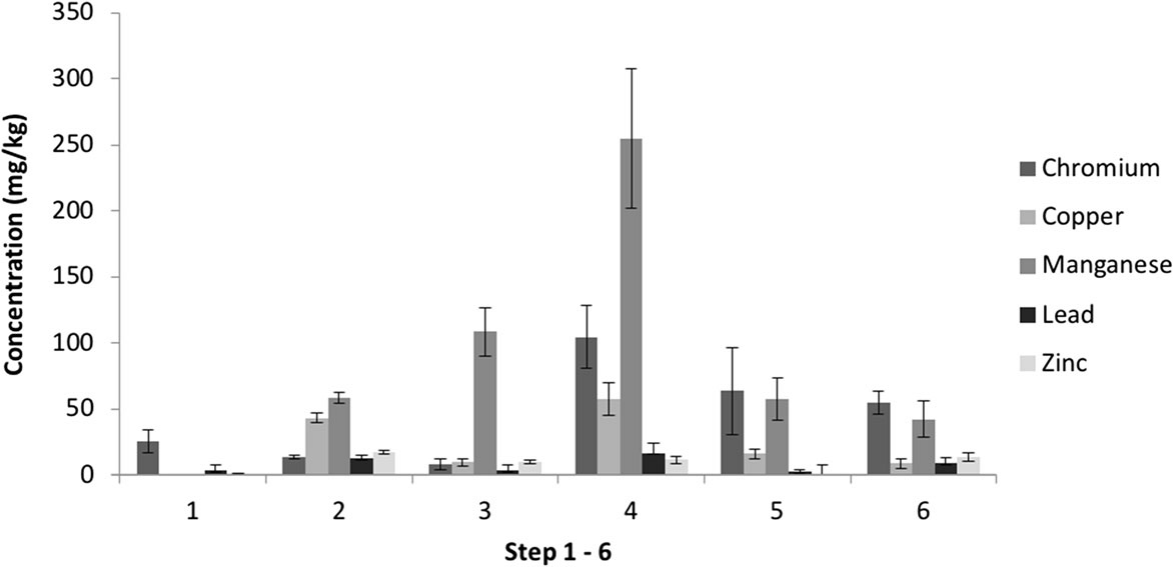
Variations between PTE concentrations from the SE extracts of flue dust samples (1—water soluble, 2—ion exchangeable, 3—carbonates, 4—Fe–Mn oxides (amorphous), 5—Fe–Mn oxides (crystalline), 6—sulphides)

**Fig. 6:**
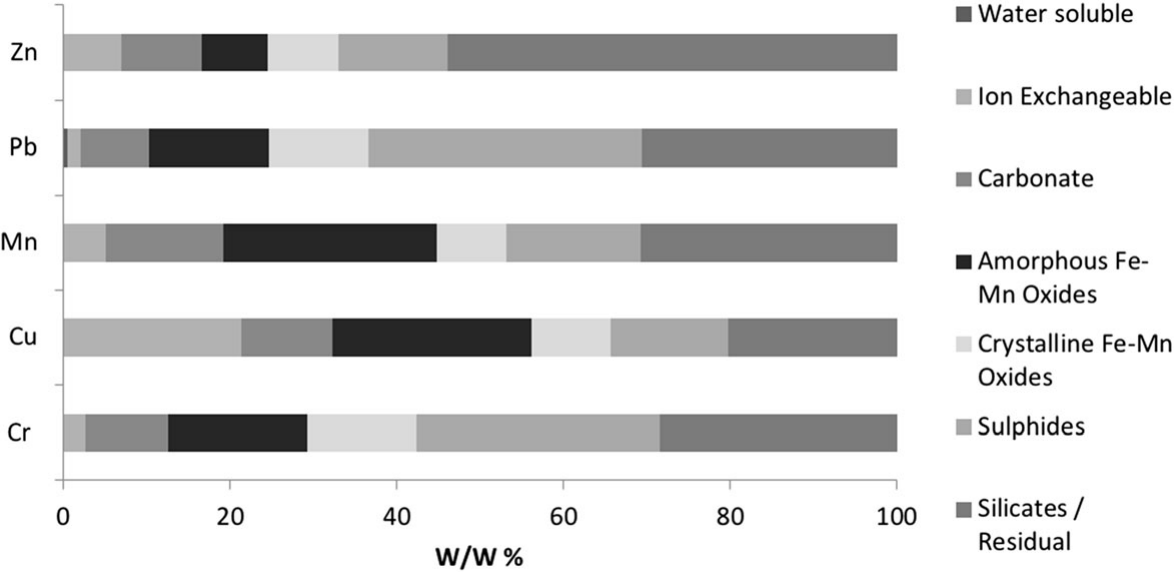
Measured elemental distribution of PTEs found in filter cake samples. Pseudo-totals: Zn 37,471 mg/kg, Pb 2010 mg/kg, Mn 4688 mg/kg, Cu 702.4 mg/kg, Cr 22.60 mg/kg

**Fig. 7:**
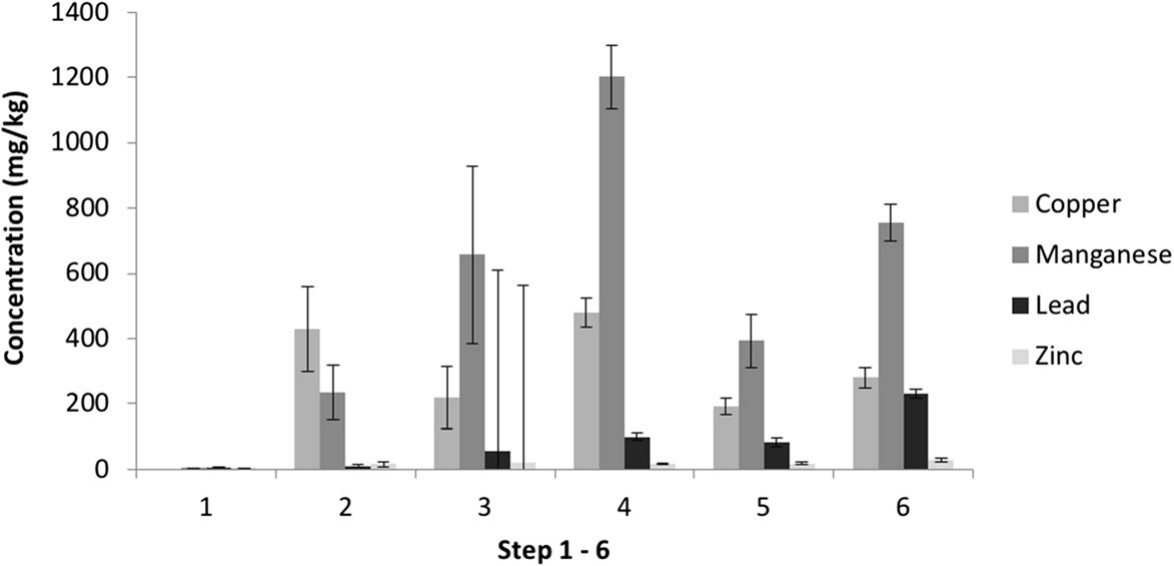
Variations between PTE concentrations from the SE extracts of filter cake samples (1—water soluble, 2—ion exchangeable, 3—carbonates, 4—Fe–Mn oxide amorphous, 5—Fe–Mn oxides crystalline, 6—sulfides)

The sequential extraction procedure (SEP) (Table 1) was applied to 20 replicates of a certified reference material (CRM), flue dust (FD) and filter cake (FC) with data summarised below (Figs. 2, 3, 4, 5, 6 and 7 inclusive). We focus on the following potentially toxic elements (PTEs) of particular interest: Cr, Cu, Mn, Pb and Zn. These elements have been highlighted as key constituents that can cause problems within the steel production process as well as having the potential to pollute the surrounding environment, for example Zn and K baring carbonates can damage and weaken the lining of the blast furnace’s lining (Besta et al. 2013, 2014).

#### Certified reference material

No steel processing by-product reference materials are commercially available for SE. The use of the furnace dust CRM (EURO STANDARD ECRM-B 877/1) provides an opportunity to identify the scope of analytical method development by comparing its certified total concentrations with the cumulative pseudo-total concentrations from the SE steps. Figure 2 shows the distribution of the key PTEs (Cr, Cu, Mn, Pb and Zn) across the various fractions, and Fig. 3 shows the variation between replicates. There was insufficient solid residue left to perform a digest of the final residual fractions but the cumulative total of the six measured extracts resulted in recoveries > 80% (excluding Cr with 66%).

The SE analysis of PTEs in the CRM extracts show the reproducibility of replicates for each extraction step (1–6 refers to ion exchangeable to sulphides). For example, fractions 3–6 show variability in concentration of less than 10%. This is similar to the pseudo-total analysis with 6–9% variation, which shows good reproducibility for the SE procedure. The first two extracts show higher variability, which is a consequence of low concentrations, i.e. close to, limit of detection (see above).

#### Flue dust

Results from the SEP applied to flue dust samples are shown in Fig. 4, with variability shown in Fig. 5. The FD samples vary most in the 1st fraction 32–189% RSD, which is symptomatic of the low concentrations and variable dissolution of these PTEs in weak reagents. The latter fractions (4–6) demonstrate higher PTE concentrations, with lower variation (4th 7–13%, 5th 14–27% and 6th 15–40%). There is a significantly large variation observed with regard to Pb and Zn found in the carbonate phase (196 and 225% respectively).

#### Filter cake

Results from sequential extraction applied to filter cake samples are seen in Fig. 6 with variability shown in Fig. 7. The distribution of PTEs shows the predominant fraction for each PTE to be different; sulphides are the main phase associated with Cr and Pb (29% and 33%), Cu and Mn are predominantly linked to amorphous Fe–Mn oxides (24% and 26%) and finally the majority of Zn can be found in the residual fraction (54%).

The filter cake samples have a much higher variation between replicate samples, which is likely to be a result of the high variation in production feed, i.e. scrap steel. The carbonate phase shows the highest variation between replicates with RSD values ranging from 40% for Mn up to 225% for Zn. This is likely to be indicative of the heterogeneous nature within the sample matrix and a variation in extraction success. The latter three fractions show a lower variation (%RSD) but still notable to suggest a significant variation in replicates: Amorphous Fe–Mn oxides varies between 7 and 13%, crystalline Fe–Mn oxides 14–27% and sulphides 16–39%. As a result, there is no evidence to suggest that the concentration of the sample influences the variation.

The model proposed for mineralogical hosts for the PTE is “probed” using routine solid-phase analysis techniques, and from the SEP results, there are a number of factors that need to be considered before assessing the success of extractions: The quantity of the final solid residuum (“residual fraction”) shown in these results has been calculated by mass balance, which means that the concentrations of dissolved species in the first six extracts have been summed and subtracted from the pseudo-total concentration. As opposed to quoting the measured content from the residual step, for two reasons:

1. The results calculated and presented here do not take into account the sample loss that takes place between each SE step as a result of the transport and filtration processes but assumes the mass measured at the start. This means all of the extracted residues, particularly the final residuum, will have lower mass compared to their original weight, e.g. 0.2 g as opposed to 0.25 g, which will result in a more conservative value obtained. This is a limitation common to all extraction procedures and can be minimised by good laboratory practice.
2. As a result of the larger variations found within the pseudo-total digestions (9–20% in flue dust replicates and 16% in filter cake), the higher uncertainty generated in data means values can be either overly conservative or liberal.

Calculation of the residual content by difference compounds these uncertainties and should not be confused with the calculation used to express the individual distribution of each PTE. This involves their respective concentrations (mg/kg) as a percent of the sample pseudo-total value as an “% w/w extracted”. The high levels of calcite and portlandite in FD and the Fe-rich composition of FC give rise for recover concerns; however, this approach proves successful dissolution with its total recovery.

Levels of chromium measured released from the sulphide fraction are also attributed to the sodium chromate that is added as an extracting reagent during this step. It is unlikely that there is Cr_2_S_3_ in the steel wastes, particularly as initial characterisation was unable to identify the presence of this phase. However, whilst other oxidisable fractions could be present in the sample, they cannot be quantified due to masking by this exaction reagent.

### Solid-phase analysis

To monitor structural changes at a molecular level between extraction steps, XRD, FTIR and SEM were used to analyse FD and FC solid residues, to identify, where possible, the effect of each extraction step during the sequential extraction process. The techniques predominantly identify changes (losses) of particular features, i.e. functional groups or crystalline compounds that in-turn can be interpreted as phase losses. It is important to identify the use of “simple” and “common” analytical methods in this evaluation, making the technique more practical in application during waste management. Alternative approaches such as use of synchrotron radiation probes are acknowledged, but not in scope for this study.

#### Powder X-ray diffraction

Initial P-XRD analysis of both FD and FC sample characterisations was carried out using QualX-software using a polynomial to estimate the background and smoothing requirements as well as SEM elemental analysis for compositional filtering and initial mineralogical comparisons.

**Fig. 8:**
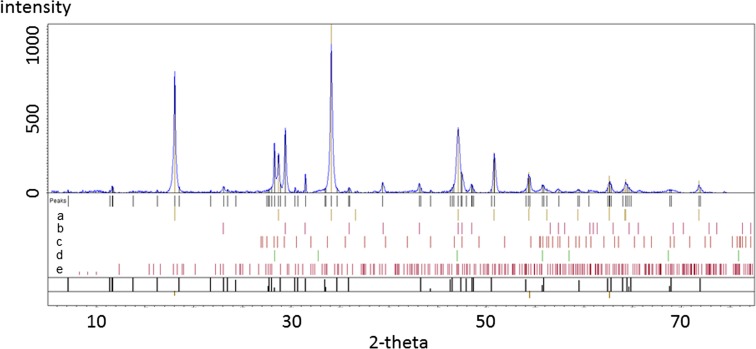
Flue dust (FD) sample. Contains a. portlandite (Ca(OH)2), b. calcite, c. wurtzite, d. fluorite, e. ettringite

**Fig. 9:**
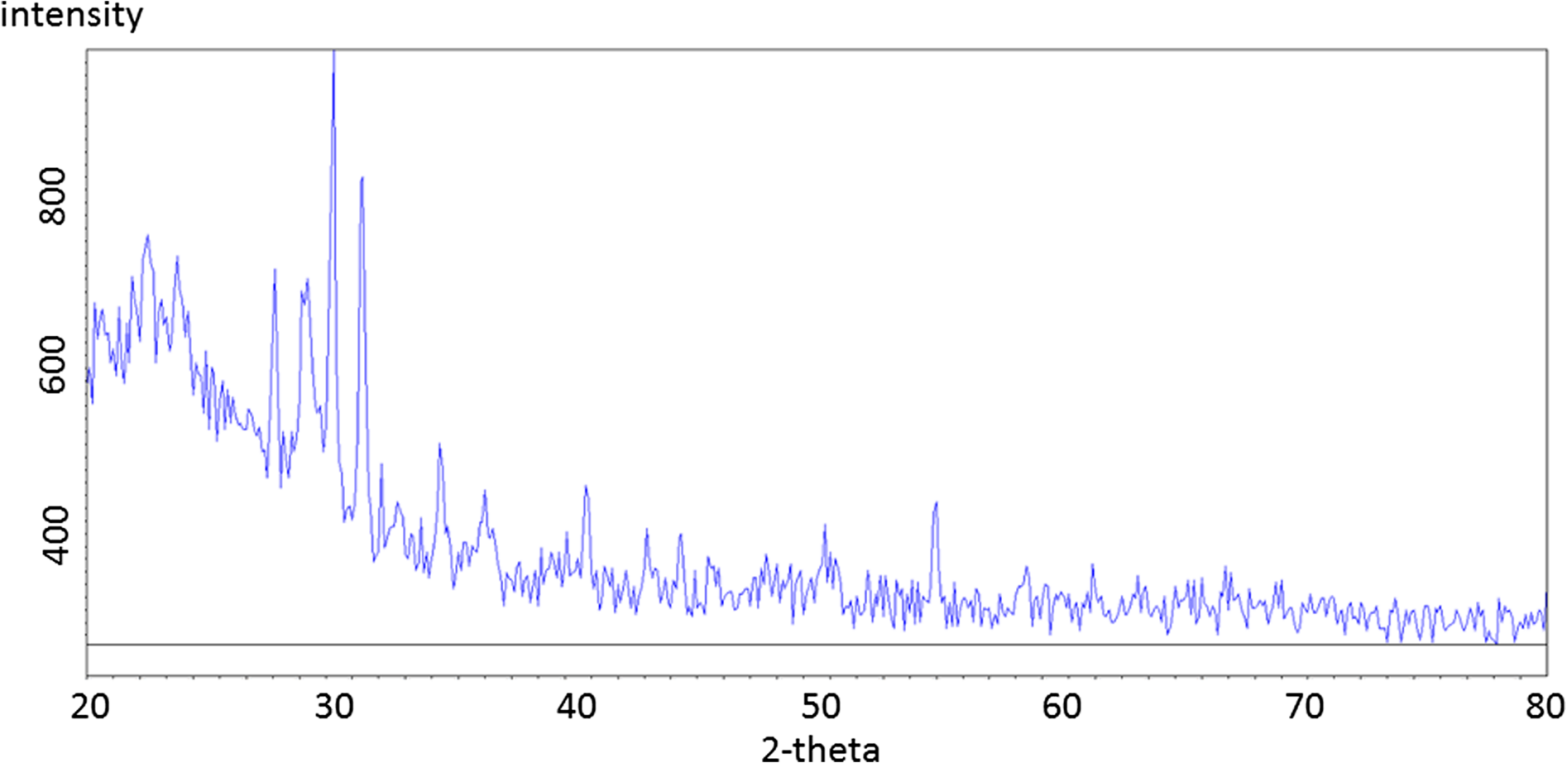
Residual flue dust (FD). Containing sodium chlorate and Ca-aluminate, the latter being the only likely residue from the starting material

The mineralogical identifications made from the FD raw and residual fraction samples (the 6th extract) are shown in Figs. 8 and 9, for comparison (diffractograms from other steps are not included). The description of the phases identified between each extract can be found below, and some spectral lines are highlighted.

The flue dust (Fig. 8), d-traces (with Kα_2_ stripped out), shows a hump at the low two-theta end indicating a substantial amount of glass. The QualX-software matching process identified major phases to be portlandite and calcite, with a few percent fluorite, a calcium aluminate phase, chromite and another Fe oxide and glass—which is likely to be calc-silicate “slag” material, e.g. Ettringite (Mindat.org 1993–2016; RRUFF 2016). This can be compared to the residual spectrum (Fig. 9) that firstly shows much lower intensities for those peaks present and no identified phases observed.

By using this analysis to follow each extraction step, it was found that fluorite disappears after the 1st extract, the removal of Calcite was observed after the 3rd extraction and the amorphous and crystalline Fe–Mn oxides were not detectable in this process and therefore cannot be confirmed. The loss of sphalerite after the extraction step 6 was also observed, confirming successful extraction of sulphide phases.

**Fig. 10:**
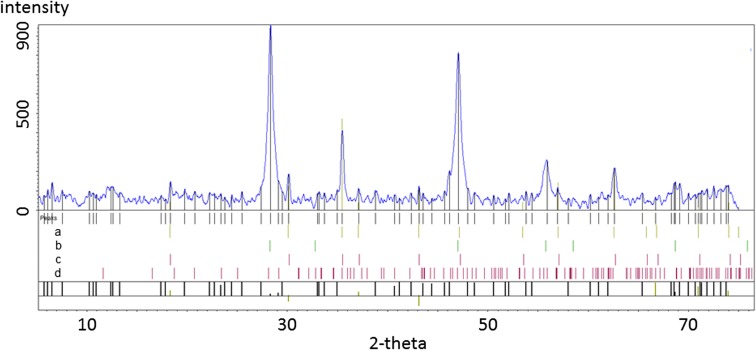
Filter cake (FC) sample. Contains a. magnetite, b. fluorite, c. chromite, d. gypsum

**Fig. 11:**
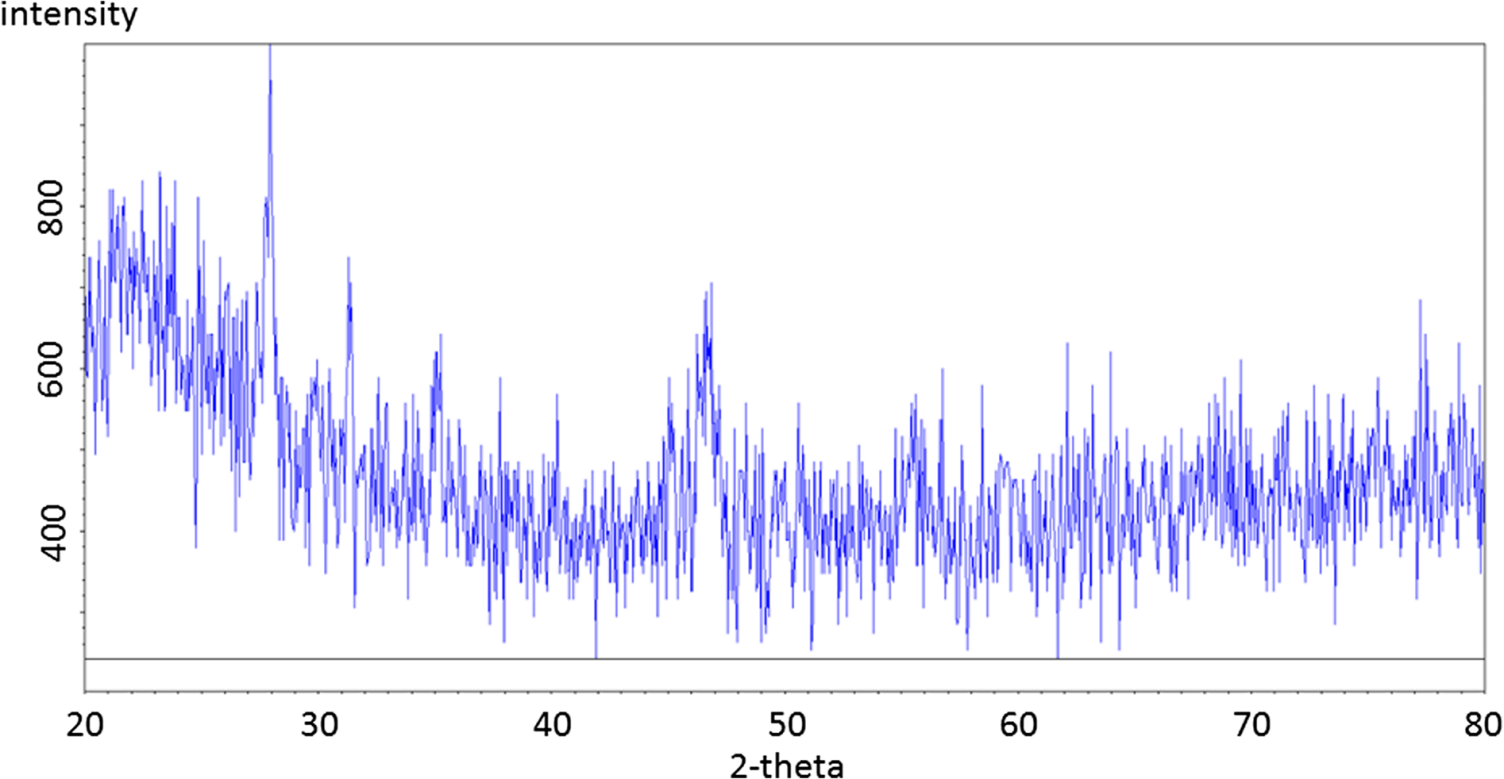
Residual filter cake (FC). Containing only silicates and oxides

The mineralogical identifications made from the raw filter cake (FC) and extraction residue fraction (taken after the 6th extract) are shown in Figs. 10 and 11.

As with flue dust, FC samples were prepared and analysed using PXRD, and the QualX software identified major phases to be fluorite and iron oxides or hydroxides, with chromitic spinels and low percentages of Ca-sulphate and Ni-sulphides. The chromite fragments are aggregates with either pores or an interstitial Ca phase, most likely calc-silicate glass.

When looking at the solid residues from separate extracts, the success of the extraction procedures was confirmed by changes in the phases identified in material from each step.

The Fe-oxide “magnetite” identified as present in the first four extracts, but after the crystalline oxide extraction (step 4), it was no longer detected. This is also observed with the loss of “sphalerite” after the sulphide extraction step 6. Although there are relatively few phases directly identified by XRD analysis, the loss of components from the diffraction pattern with peak assignments, demonstrates that the impact of extraction agents on the solid phases follows protocol as designed. However, it is important to remember that their complete dissolution cannot be confirmed due to the limit of detection (~ 3%) for this analytical method (P-XRD).

#### Scanning electron microscopy–energy-dispersive X-ray spectroscopy

SEM-EDX (energy-dispersive X-ray spectroscopy) analysis was performed on the residues of the various extracts for both FD and FC samples using a “gunshot residue approach” (Zeichner and Levin 1993); this approach simply uses tape that residual powder sticks too. The residual flue dust samples were too difficult to analyse due to their fine particulate, small quantity and its nature to be easily charged and subsequently lost in analysis.

### Flue dust

**Fig. 12:**
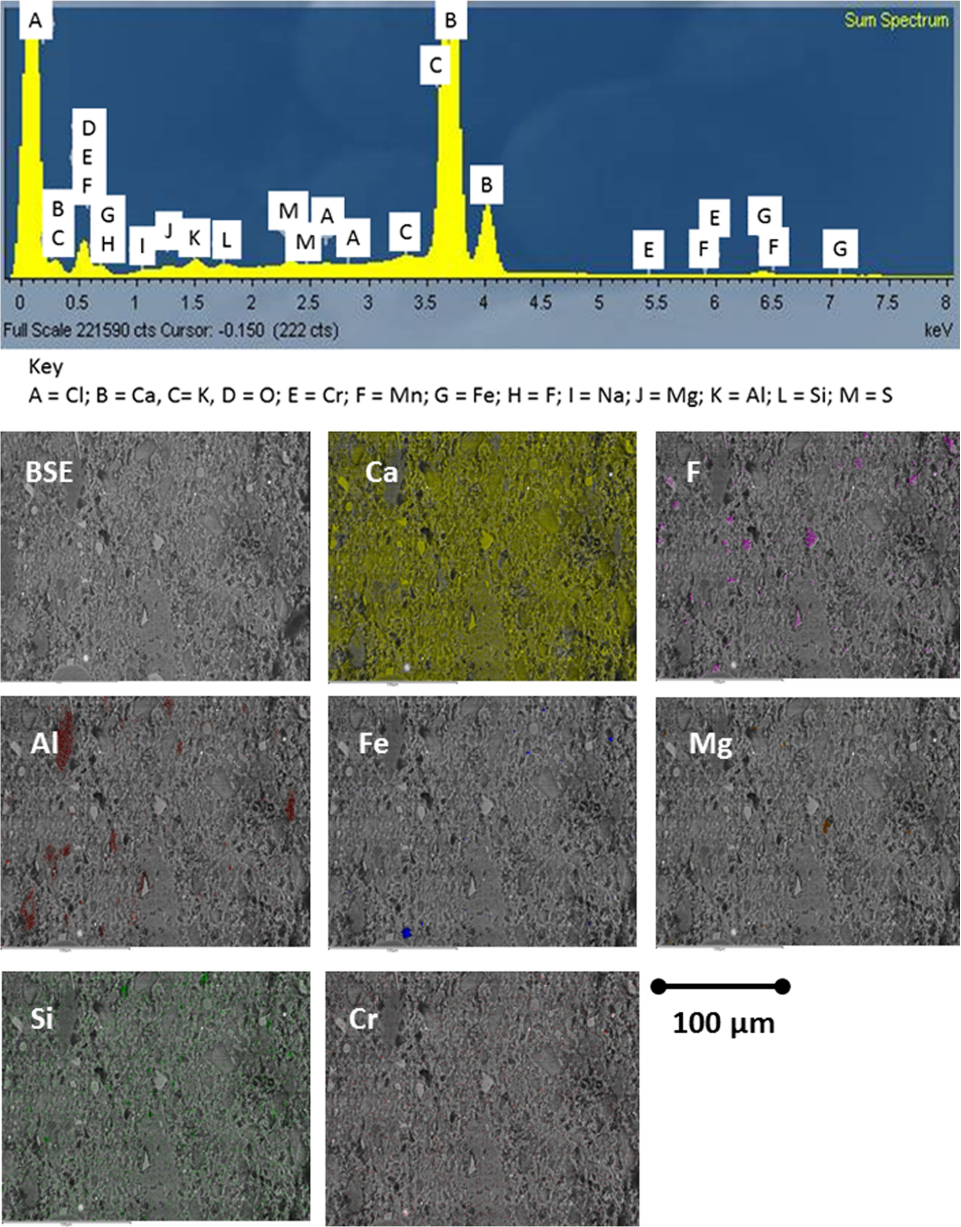
SEM EDX spectrum and elemental maps of raw FD sample

A general background was determined for FD residues using SEM-EDX (Fig. 12). A wide-area sum spectrum shows one very large peak for Ca with the next largest for O, with possible minor peaks for Al, F, Mg, Na, S, Si, Fe, K and Cl. EDX maps show that Ca is present throughout most of the sample; it has much lower pixel density in some larger, elongated, irregular-shaped particles that are enriched in Al, as well as seen in calcium silicate particles. The pale grey angular fragments and rare ovoid pale objects are a Ca + F compound (fluorite CaF_2_) and small, BSE-bright particles indicative of Fe with some rich in Cr.

It can be inferred that the finer particulate offers a greater homogenous mixture as sulphur can be seen in its background but not in the filter cake. It can be concluded that larger sample sizes would be needed in order to utilise SEM as an applicable technique for its analysis.

### Filter cake

**Fig. 13:**
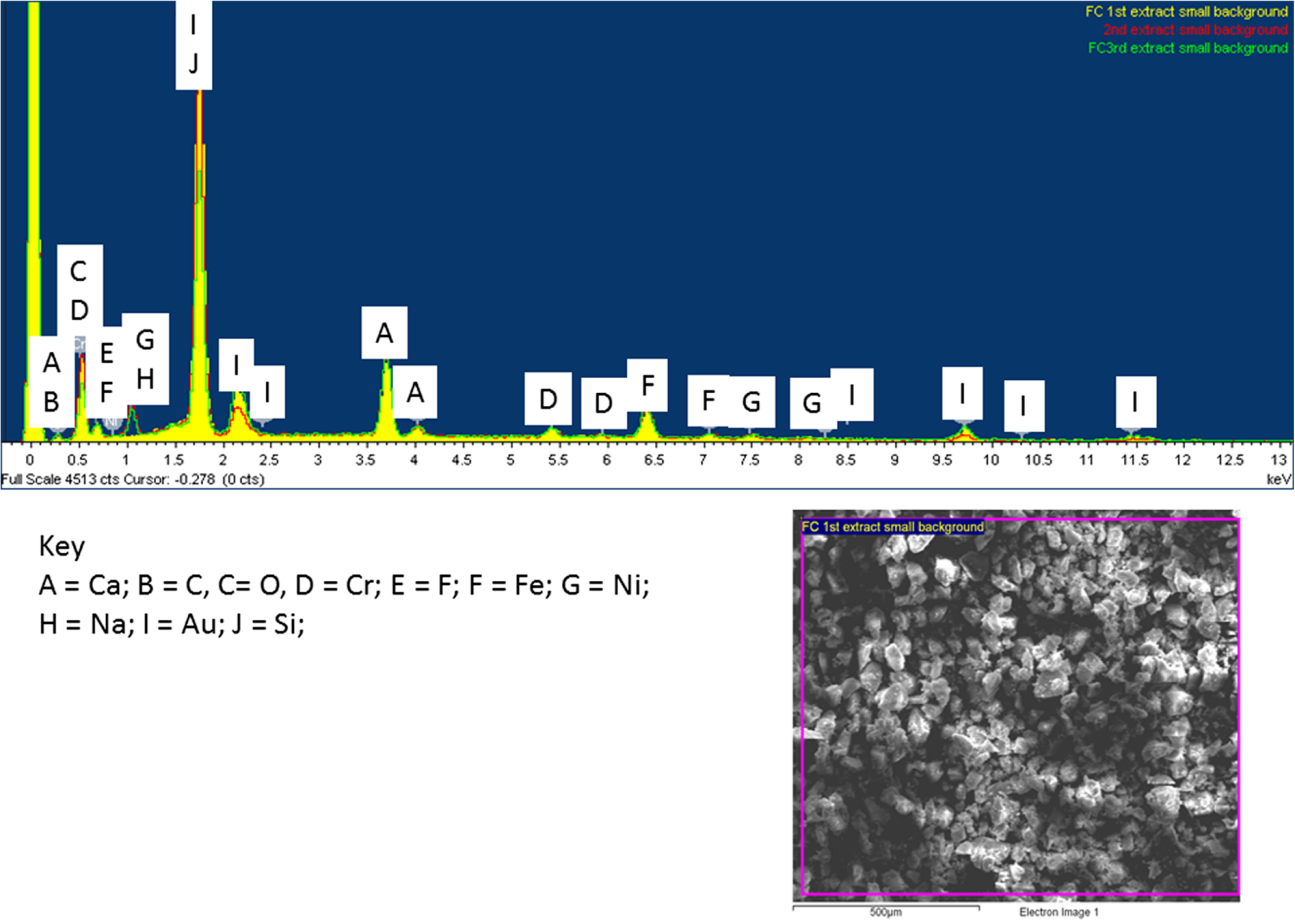
SEM EDX spectra of FC residues from the first three extracts with backscatter image of bulk sample

**Fig. 14:**
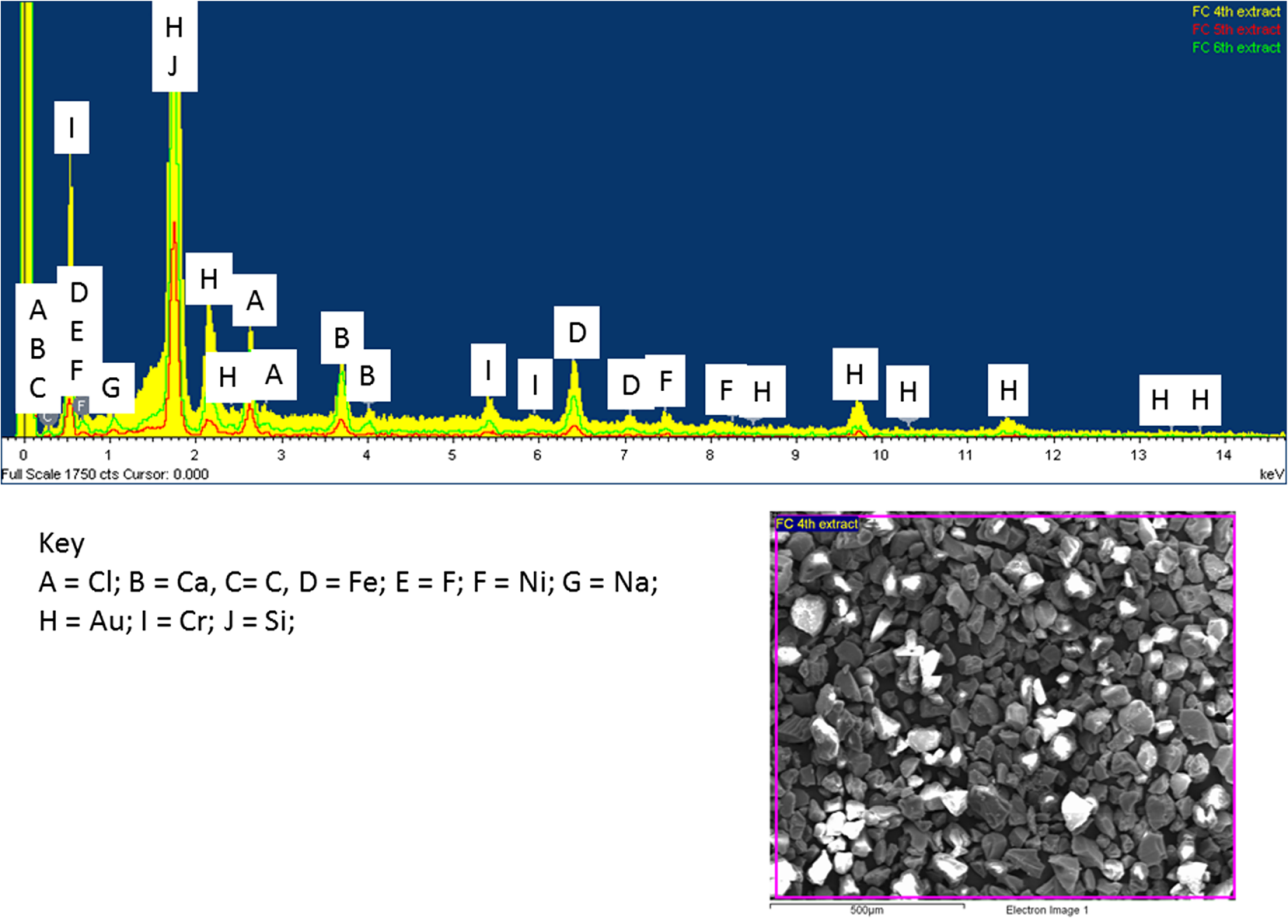
SEM EDX spectra of FC residues from the 4th, 5th and 6th extracts with backscatter image of bulk sample

The filter cake general background for all the extracts have been determined and shown in Figs. 13 and 14. Here for the first three extracts, it can be observed that the amount of silicon decreases along with the oxygen throughout the extraction steps (Fig. 13); this reduction can be attributed to the initial addition of Si gel from the first step, whereas the consistent peak after step 3 is likely to be representative of sample silica. It can also been observed that Na appears in the second two extracts as a result of the sodium acetate extraction reagent. The overall elemental matrix shows to be consistent throughout.

The 4th, 5th and 6th extracts are where more noticeable differences occur (Fig. 14). All samples where run in the same session; therefore, the intensity of these peaks can be comparable in terms of concentration inferences. Therefore, it can be stated that the general background remains the same but decreases in concentration chronologically. It is worth nothing that spot analysis was carried out on each extract with sulphur being found with the exception of the 6th extract, which is indicative of successful removal of sulphides.

### Fourier transform infrared spectroscopy

FTIR was used to identify specific functional groups/bonding present in the samples that may be characteristic of the phases being “extracted”. For example the removal of carbonates would be seen by the reduction of the C–O and C=O signals. By re-evaluating after each extraction has taken place, it can also be used as a confirmatory tool to the success of the extraction procedure and specificity of the reagents used. It is also important to recognise that FTIR identifies bond relationships and cannot identify specific phases, adding information to the development of phase model for the samples. It has been used widely in inorganic and mineralogical analysis (Chen et al. 2015). The analysis of the FTIR was between 600 and 4000 cm^−1^; however, the transmittance for both FD and FC samples does not alter after 2000 cm^−1^ and therefore is not shown here.

### Flue dust

**Fig. 15:**
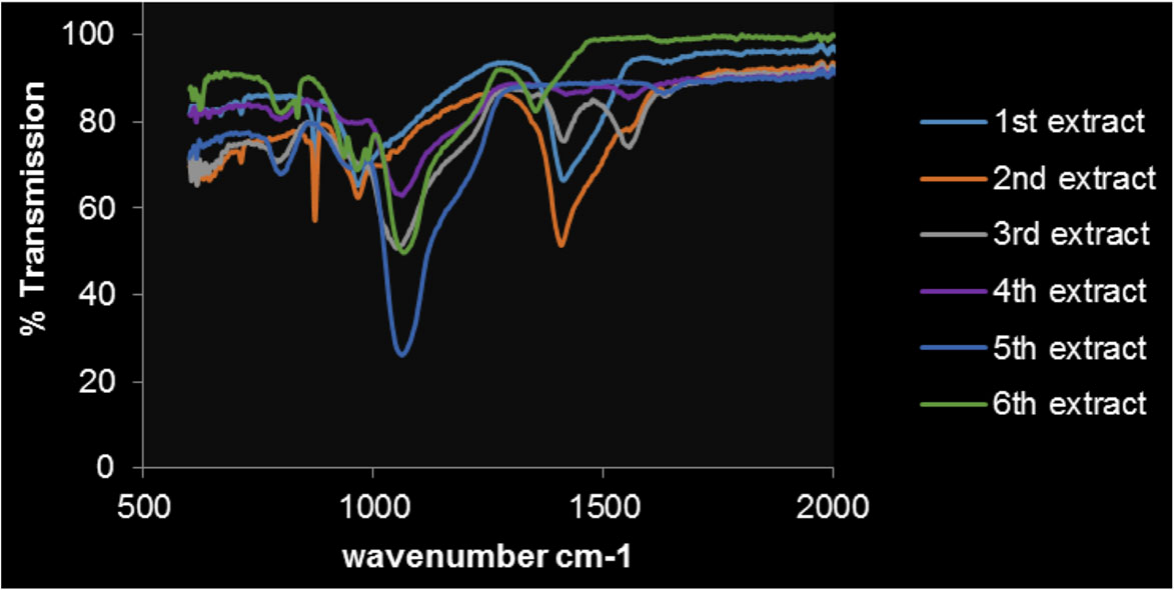
FTIR of flue dust after each of the six extractions

The residues from each sequential extraction step were air-dried and subjected to FTIR analysis; the corresponding spectra obtained are seen in Fig. 15. It shows the changes in transmittance after each extract where 1–6 refers to the different extractions, i.e. 1—water soluble, 2—ion exchangeable, 3—carbonate, 4—Fe oxides (amorphous), 5—Fe oxides (crystalline) and 6—sulfides.

It can be noted in Fig. 15 that clear trends are harder to identify as each extraction takes place. The initial extracts show a variety of C–H and C–C stretching bands (1420 cm^−1^ CH_2_; 1566 cm^−1^ C–C), which could be linked to coke added during the steel production process. The addition of sodium acetate and acetic acid, as extraction reagents, may have added signal to the IR spectrum through contamination as samples were not washed between steps to minimise losses. They were noticeably absent after the 4th extract, i.e. when organic reagents were no longer being used.

The observed peak at 1050 cm^−1^ could be indicative of C–O primary bonds or additional C–C bands, which can be linked to graphite (National institute of Standards and technology 2016) and consequently coke (Speight 2015). This C-based complex reacts with oxygen under a gas–solid reaction that occurs with two distinct steps: adsorption of molecular oxygen and surface diffusion of the oxygen molecules. These reactions can be seen below: There are numerous complex reactions between oxygen and carbon, depending on the temperature regime and stage of the batch process giving rise to changes in the dust composition.

Graphite undergoes this reduction to form species such as O_2_^−^, or O_2_^2−^ until it finally makes stable covalent bonds with carbon atoms (Ulbricht et al. 2002). Due to the low reactivity of graphite, this can explain its lack of removal before the final digestion step and continuous C–O liberation and subsequent identification.

The combination of peaks at 1050 cm^−1^, 1200 cm^−1^ and 800 cm^−1^ (which can be seen from the 5th and 6th extracts) is indicative of silica containing complexes (Launer and Arkles 2013; Smith 1960; Sayed and Zeedan 2012; Müller et al. 2012) and would explain their stability during extraction. The glassy matrix identified in the P-XRD is likely to contain Si phases, whilst not directly identified by distinct diffraction lines. These bond associations can be seen after the removal of more prominent peaks, i.e. CH_2_ from the first two extracts. Other spectroscopic technique such as Raman spectroscopy might reveal fingerprint details and allow interpretation for Si and metal containing compounds. For example for absorbance at 540 cm^−1^, the confirmation of Si–O–Si (quartz) could be made (Balachandran 2014). The sharp absorption in the FTIR observed around 870 cm^−1^ and smaller peaks around 800-850 cm^−1^ are also indicative of oxides, which can be seen to reduce in intensity over the extraction steps.

The FTIR analysis does show that the extraction of the carbonate phases and potentially the sulphide phases is successful for both sample types.

### Filter cake

**Fig. 16:**
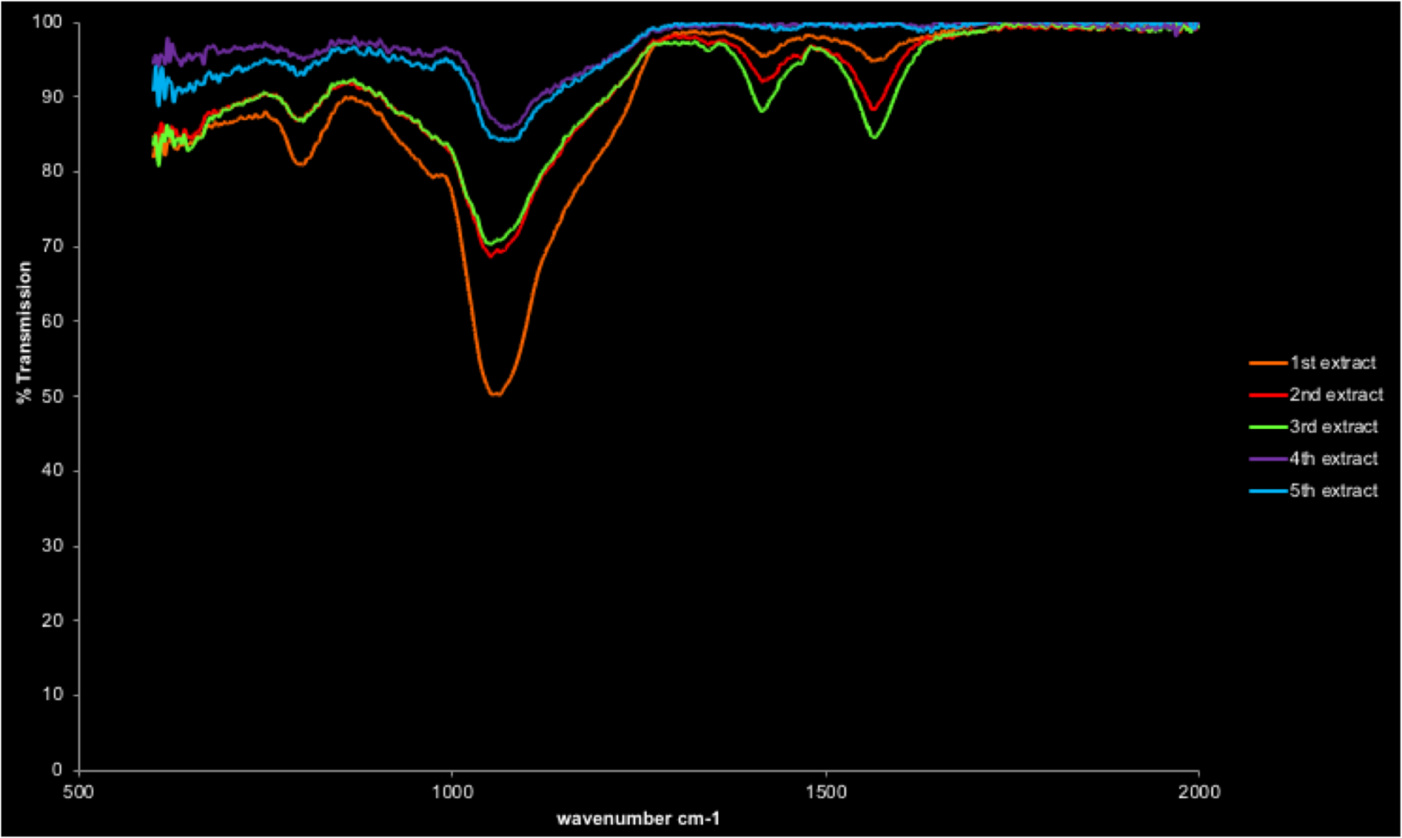
FTIR of filter cake after each of the first five extractions

Figure 16 shows the changes in transmittance as the filter cake sample is extracted, note that the sulphide (6th) extract is not shown as 100% present due to no transmittance was observed being detected. The peaks at 1566 cm^−1^ and 1418 cm^−1^, again, are indicative of the addition of extraction reagents sodium acetate and acetic acid. The peak around 1050 cm^−1^ indicates primary CO bonds, similar to that from the FD sample. Its absence in the 6th extract suggests dissolution is complete.

This is also the case for the 540 cm^−1^ Si–O–Si stretch for quartz, as although the corresponding peak cannot be measured, unlike the FD samples, its presence disappears by the 6th extract. It is possible this continuous peak represents a SO sulfoxide bond, and confirmation peaks would be found in the “fingerprint” region (below 600 cm^−1^) and therefore are difficult to confirm using this type of analysis. This peak is observed to reduce over the initial extraction steps but disappears by the 6th extract. The sulfoxide signatures are likely to be derived from the oxidation of sulphides, and their presence could be a result of desulphurisation during steel production and sulfide oxidation by the extraction reagents. The bonds are short-range bonds (Pauling 1948; Calligaris 2004), with pseudo-crystalline structures and unable to be identified with the other analytical techniques. The weak peak around the 800 cm^−1^ region is indicative of the presence of oxides and decreases throughout the extraction steps as the sample matrix is decomposed.

The solid-phase analysis (SPA) performed on sacrificial residual solid samples from SE steps was able to highlight the removal of some phases: carbonates specifically using FTIR analysis and sulphides using XRD and SEM in FC samples. XRD analysis also confirms the successful removal of crystalline Fe–Mn oxide.

## Conclusions

This study has confirmed that the application of a seven-step sequential extraction method for steel industry process by-products provides phase association data for potentially toxic elements of environmental and process concern. It is able to selectively extract important host phases and can allow an analyst to characterise potentially toxic elements within the waste gaining more detailed knowledge of the reactivity and potential classification. There are however a number of considerations and recommendations:

1. The waste material used in the assessment (flue dust and filter cake) are inherently fine grained and the results have shown a high variability between extraction replicates. It is believed that this is most likely to be caused by the extreme distribution of elements between component phases within the material (i.e. some oxide, metallic phases, carbonate and sulphide components. The heterogeneous nature is difficult to overcome, and the analyst must take this into consideration for industrial application the maximum sample size, determined by vessel volumes to preserve extraction solid/solution ratios, that needs to be used.
2. The traditional BCR procedure for environmental matrices involves three overnight shaking periods to ensure that the reaction is complete (Bacon and Davidson 2008). The procedure detailed in this paper have been modified and adapted from previous studies (Leinz et al. 2000) with shorter contact times (0.5–2 h). Whilst this is “short” for liquid solid reactivity, it is within the legislative requirements of exposure time being no more than 24 h. In addition, the conditions used within this experiment (e.g. concentrated acids and bases) are not what would be encountered in under “real-life” exposure, i.e. acid rain leaching. It is our recommendation therefore that short exposure times are used; however, the analyst must consider that a small fluctuation, e.g. 5–10 min at 30-min exposure, represents an additional 20–30% increase in contact time and subsequent influence on elemental extraction.
3. It has been shown that sequential extraction (SE) can successfully apportion elements to specific phases. If this is combined with the pseudo-total data, then fuller predictions can be made about the elemental composition of steel waste. For example, Cr in the filter cake had a total concentration of approx. 26,000 mg/kg with 14.4% found within the carbonate (3rd step) most likely as Cr_2_(CO_3_)_3_. Although SE does not give a complete mineralogical identification, it provides sufficient information about an element’s most likely host phase allowing factors that could be used to promote release from waste (e.g. for recovery and re-use) and/or predict environmental impact and subsequent classification as hazardous or non-hazardous waste.
4. This information could be used as a useful environmental indicator related to current Waste Acceptance Criteria (WAC) regulatory testing European Commission 2009). Using Cr as an example, the water soluble fraction (1st extract) of the filter cake had a median concentration of 9.1 mg/kg; under WAC thresholds, this would be categorised as non-hazardous waste (even if the high replicate variation was taken into account) as it is below the 70 mg/kg threshold (Environment Agency 2013). The content in the carbonate phase however could be considered hazardous as the portion contained in this phase is reactive if introduced to the environment.

This study has shown that sequential extraction (SE) can be considered a low-cost “simple” analytical methodology when compared to the limitations of some instrumental techniques such as X-ray diffraction, Fourier transform infrared spectroscopy and scanning electron microscopy, which provide important feedback on the reactivity of process by products. The use of SE has industry wide implications for application to address concerns about definition of hazardous waste (and identify methods to treat them accordingly). This brings us a step closer to more circular resource management and implications for cost-effective regulatory systems, enhancing environmental compliance.
